# A Novel Method for Fault Diagnosis of Rotating Machinery

**DOI:** 10.3390/e24050681

**Published:** 2022-05-12

**Authors:** Meng Tang, Yaxuan Liao, Fan Luo, Xiangshun Li

**Affiliations:** School of Automation, Wuhan University of Technology, Wuhan 430070, China; tangmeng@whut.edu.cn (M.T.); lyx19971020@whut.edu.cn (Y.L.); lixiangshun@whut.edu.cn (X.L.)

**Keywords:** fast iterative filtering, parameter adaptive refined composite multiscale fluctuation-based dispersion entropy, rotating machinery, fault diagnosis

## Abstract

When rotating machinery fails, the consequent vibration signal contains rich fault feature information. However, the vibration signal bears the characteristics of nonlinearity and nonstationarity, and is easily disturbed by noise, thus it may be difficult to accurately extract hidden fault features. To extract effective fault features from the collected vibration signals and improve the diagnostic accuracy of weak faults, a novel method for fault diagnosis of rotating machinery is proposed. The new method is based on Fast Iterative Filtering (FIF) and Parameter Adaptive Refined Composite Multiscale Fluctuation-based Dispersion Entropy (PARCMFDE). Firstly, the collected original vibration signal is decomposed by FIF to obtain a series of intrinsic mode functions (IMFs), and the IMFs with a large correlation coefficient are selected for reconstruction. Then, a PARCMFDE is proposed for fault feature extraction, where its embedding dimension and class number are determined by Genetic Algorithm (GA). Finally, the extracted fault features are input into Fuzzy C-Means (FCM) to classify different states of rotating machinery. The experimental results show that the proposed method can accurately extract weak fault features and realize reliable fault diagnosis of rotating machinery.

## 1. Introduction

Rotating machinery, such as electric motors, centrifugal pumps, and turbine engines, represent the most widely used mechanical equipment in industrial processes [1]. The mechanical equipment usually operate under unstable loads and harsh working conditions, thus various failures of their critical components, such as bearing damage and impeller damage, are inevitable. The operating states of rotating machinery directly affect the productivity and safety of the industrial sector. Therefore, accurate and reliable fault diagnosis of rotating machinery is of great practical significance [2].

The key to fault diagnosis of rotating machinery is to extract fault features from vibration signals. Vibration signals are nonlinear and nonstationary [3], and are easily interfered by noise, thus it is difficult to extract hidden features. Therefore, it is necessary to combine the appropriate time–frequency analysis method with the entropy measurement method to extract the hidden tiny fault features. The first step is to choose the appropriate signal processing method. Studies have shown that when the fault signal is disturbed by noise, traditional time–frequency analysis techniques, such as Fourier transform (FFT) and Wavelet Transform (WT) cannot accurately extract fault features [4,5]. The more commonly used method is the Empirical Mode Decomposition (EMD) method proposed by Huang et al. in 1998 [6]. The EMD can adaptively decompose the signal into the sum of finite intrinsic mode functions (*IMF*), each *IMF* component represents a set of characteristic scale signals, and the feature extraction of each component can better reveal the fault information intrinsic characteristics. However, EMD suffers from modal aliasing, end-point effects, and a lack of rigorous mathematical framework for using envelopes in an iterative manner [7]. Although the Ensemble Empirical Mode Decomposition (EEMD) [8] optimized on the basis of EMD can effectively improve the problem of mode aliasing, and the Fast Ensemble Empirical Mode Decomposition (FEEMD) [9] further improves the calculation speed, neither escape the drawbacks of using envelopes in an iterative fashion without a rigorous mathematical framework. Subsequently, Dragomiretskiy K et al. proposed a new adaptive Variational Mode Decomposition (VMD) method. The method is a non-recursive variational decomposition model, and the optimal solution of the variational model is iteratively searched by the alternating direction multiplier method, thereby determining the center frequency and bandwidth of each mode. It avoids mode mixing in EMD, and has better robustness to noise [10]. However, VMD suffers from relatively slow computational efficiency, and its performance depends heavily on its two input parameters, namely the penalty factor and the number of decomposition modes [11]. The Iterative Filtering (IF) method proposed by Lin et al. and its derivatives [12], such as the Adaptive Local Iterative Filtering (ALIF) method [13], the Fast Iterative Filtering (FIF) method [14] can produce results similar to EMD-based algorithms, with the important advantage that their convergence and stability are guaranteed. Moreover, the FIF method uses a fixed low-pass filter function to replace the envelope mean curve in the EMD method, which solves the problem of EMD lacking a strict mathematical framework. Meanwhile, the FIF method is unaffected by mode aliasing, and mode splitting can be easily avoided by adjusting the value of the stopping criterion parameter [4]. Furthermore, FIF greatly improves the calculation speed on the basis of ensuring decomposition accuracy, with small decomposition error, good noise robustness, and can achieve efficient and accurate signal decomposition [15]. Therefore, this paper adopts the FIF method to decompose the vibration signal of rotating machinery.

The components of the vibration signal following decomposition by FIF contain rich fault information. Moreover, the components of vibration signals in different states of rotating machinery show different complexity, so the entropy parameter can be used to extract the fault information [16]. Approximate Entropy (ApEn), Sample Entropy (SampEn), Fuzzy Entropy (FE), and Permutation Entropy (PE) are widely used in the field of rotating machinery fault diagnosis to measure the complexity of vibration signals [17,18,19]. However, ApEn and Multiscale Approximate Entropy include the comparison of their own data segments in the calculation process, and their calculation depends on the data length. If the data length is short, the obtained value is usually smaller than the actual value. The SampEn is an improvement on the approximate entropy. It does not include the comparison of its own data segments, and has higher calculation accuracy and better consistency. However, SampEn and its improvements also have clear shortcomings: Firstly, SampEn and its improvements use Heaviside functions to measure the complexity of time series, resulting in inaccurate estimates in practical applications [20]. Secondly, SampEn and its improvements are computationally inefficient, especially for long time series. FE and its improvements replace the Heaviside function with a fuzzy membership function that is insensitive to background noise and highly sensitive to dynamic changes, but it is computationally inefficient [16]. PE is a method to measure the complexity of chaotic time series. PE has high computational efficiency, can be used to calculate huge datasets, and exhibits good anti-noise performance. However, the main disadvantage of PE is that it is prone to generating undefined entropy values for short-term time series and cannot classify well-defined patterns for a specific design [21]. In order to overcome the above problems, Hamed Azami et al. proposed a nonlinear time complexity evaluation method of Dispersion Entropy (DE). DE can generate reliable entropy values, is insensitive to noise interference, can accurately capture signal characteristics, and calculate with high efficiency [22]. Subsequently, in order to improve the extraction ability of hidden fault features, Hamed Azam et al. continued to propose the Refined Composite Multiscale Fluctuation-based Dispersion Entropy (RCMFDE), which can more accurately analyze the complexity of nonlinear time series under various scale factors, with more stable entropy values [23].

However, in the RCMFDE method, there are two key parameters (i.e., embedding dimension and class number) that need to be manually selected in advance. Furthermore, the parameter setting of the RCMFDE algorithm will affect the final processing result. If the parameter settings are unreasonable, the hidden tiny fault features may not be accurately extracted, resulting in misclassification. Aiming at the determination of the embedding dimension m and the class number c in the RCMFDE algorithm, this paper proposes a Parameter Adaptive Refined Composite Multiscale Fluctuation-based Dispersion Entropy (PARCMFDE). The method takes skewness as the objective function, and uses a Genetic Algorithm (GA) to optimize parameters of RCMFDE. PARCMFDE can automatically and effectively determine the important parameters of RCMFDE, so as to describe the complexity and uncertainty of time series more accurately, and achieve the purpose of extracting the features of hidden faults. In view of the shortcomings of existing methods, relevant research is carried out, and the main contributions are as follows:

(1) PARCMFDE based on GA is proposed, which overcomes the insufficiency of experience-based parameter selection. PARCMFDE can more accurately extract tiny fault features hidden in vibration signals of rotating machinery.
(2) A fault diagnosis method for rotating machinery based on FIF, PARCMFDE and Fuzzy C-Means (FCM) is proposed, which can classify rotating machinery faults accurately and automatically without depending on the length of data samples.
(3) The effectiveness of the method is verified by the bearing data of Case Western Reserve University and the experimental data of centrifugal pumps obtained by building a water circulation experimental system. Compared with other methods, it shows that feature extraction of PARCMFDE is more accurate and stable, and the rotating machinery fault diagnosis method based on FIF, PARCMFDE and FCM exhibits better classification effect.

This paper is mainly divided into the following sections: Section 2 briefly introduces the basic principles and characteristics of the FIF algorithm. In Section 3, the principle of PARCMFDE is introduced and compared with RCMFDE and Multiscale Sample Entropy (MSE) and Multiscale Fluctuation-based Dispersion Entropy (MFDE). Section 4 briefly introduces the principle and evaluation index of FCM. Section 5 presents the method of fault diagnosis of rotating machinery. Section 6 verifies the effectiveness of the method and compares it with other vibration signal fault diagnosis methods through the bearing data of Case Western Reserve University and experimental data from centrifugal pumps obtained by building a water circulation experimental system. Section 7 provides the conclusion.

## 2. Fast Iterative Filtering

The key idea of Fast Iterative Filtering is to iteratively subtract the simple oscillatory components contained in the signal from the signal itself, the so-called IMFs, by approximating the moving average of the signal, thereby separating the simple oscillatory components in the signal [14]. The approximate moving average is computed by convolution with the window/filter function *w*. Consider a raw vibration signal s(x), define a window/filter function *w* is a non-negative even function in the range of $C^{0}\left(\left[-L,L\right]\right),L>0$. The Fokker–Plank filter is used here, and $\int_{R}w\left(z\right)z=\int_{-L}^{L}w(z)z=1$, $\hat{s}$ denotes the Fourier transform of *s*, DFT denotes the discrete Fourier transform, and IDFT denotes the inverse discrete Fourier transform. The specific implementation process of FIF is as follows:

(1) Calculate the length *L* of the corresponding filter *w* of the signal s(x):
  (1) $$L:=2\left\lfloor \xi \frac{N}{k}\right\rfloor$$
  where *N* is the total number of sampling points of the signal s(x), *k* is the number of its extreme points, and ξ is a tuning parameter, which is usually fixed around 1.6 for the Fokker–Plank filter.
(2) Calculate the discrete Fourier transform of the signal s(x) and the corresponding filter *w*, denoted as DFT(s) and DFT(w), respectively.
(3) Calculate ${\hat{s}}_{m+1}$:
  (2) $${\hat{s}}_{m+1}=(I-diag{\left(DFT\left(w\right)\right)}^{m}DFT\left(s\right)$$
(4) Calculate $N_{0}\in N$ and IMF:
  (3) $$\frac{{N_{0}}^{N_{0}}}{{\left(N_{0}+1\right)}^{N_{0}+1}}<\frac{\delta }{\left|\right|s_{m}|{|}_{2}}$$
  (4) $$IMF=\sum_{k=0}^{n-1}u_{k}{\left(1-\lambda_{k}\right)}^{N_{0}}\sigma_{k}=IDFT\left({\left(I-D\right)}^{N_{0}}DFT\left(s\right)\right)$$
  where δ > 0, represents the required precision; $N_{0}$ represents the number of iterations required to achieve the required precision δ when calculating a specific IMF; $\sigma_{k}$ represents the *k*th element of the Fourier transform of the signal *s*; $\lambda_{k}$ represents the *k*th eigenvalue; $u_{k}$ is the *k*th eigenvector; *I* is the identity matrix; *D* is the diagonal matrix, whose diagonal is the eigenvalue.
(5) Judgment of inner loop stop condition: if the stop standard SD is met, then stop the inner loop, otherwise let m=m+1 repeat steps (3)–(5), the stop standard SD is calculated by the following formula:
  (5) $$SD:=\frac{\left|\right|s_{m+1}-s_{m}|{|}_{2}}{\left|\right|s_{m}|{|}_{2}}<\delta ,\forall m\geq N_{0}.$$
(6) Calculate the IMF component and the new *s*:
  (6) $$IMF=IMF\cup \{IDFT\left({\hat{s}}_{m}\right)\}$$
  (7) $$s=s-IDFT({\hat{s}}_{m}).$$
(7) Judgment of outer loop stop condition: Calculate the extreme point of *s*, if there is only one extreme point of *s* or less, the outer loop stops, otherwise repeat steps (1)–(7).
(8) Extract the final *IMF* component
  (8) IMF=IMF∪{s}.

In short, the FIF method includes two processes: inner loop and outer loop. The purpose of the inner loop is to filter out the *IMF* components of each order. The purpose of the outer loop is to end the process of extracting the *IMF* component of the inner loop. When the residual obtained by removing all *IMF* components from the original signal s(x) contains only one or less extreme points, the outer loop stops.

## 3. Parameter Adaptive Refined Composite Multiscale Fluctuation Based Dispersion Entropy

### 3.1. Refined Composite Multiscale Fluctuation-Based Dispersion Entropy

Refined Composite Multiscale Fluctuation-based Dispersion Entropy (RCMFDE) accounts for the shortcomings of Multiscale Fluctuation-based Dispersion Entropy (MFDE) in the process of coarse-graining, which has low computational efficiency and a high probability of invalid entropy values. The entropy value is more stable, the operation speed is faster, and the probability of invalid entropy occurrence is greatly reduced. The specific process of RCMFDE is as follows:

(1) For a given univariate signal $L:v=\{v_{1},v_{2},\cdots ,v_{L}\}$. Dividing *v* into non-overlapping segments of length τ is called the scale factor. Construct a composite coarse-grained time series:
  (9) $$x_{k}^{\left(\tau \right)}\left(i\right)=\frac{1}{\tau }\sum_{c=(i-1)\tau +k}^{i\tau +k-1}v_{c},1\leq i\leq \left\lfloor \frac{L}{\tau }\right\rfloor =n,k=1,2,\cdots ,\tau$$
  where *k* represents the coarse-grained sliding number of the scale factor under τ.
(2) Map $X=\{x_{1},x_{2},\cdots ,x_{n}\}$ to $Y=\{y_{1},y_{2},\cdots ,y_{n}\}$ through the normal cumulative distribution function (NCDF) as follows:
  (10) $$y_{k}\left(i\right)=\frac{1}{\sigma \sqrt{2\pi }}\int_{-\infty }^{x_{k}\left(i\right)}e^{\frac{-{\left(t-\mu \right)}^{2}}{2\sigma^{2}}}dt$$
  where σ is the standard deviation of the time series *X* and μ is the mean.
(3) Linearly assign $y_{k}\left(i\right)$ to an integer $z_{k}\left(i\right)$ from 1 to *c* as follows:
  (11) $$z_{k}^{c}\left(i\right)=round\left(c\times y_{k}\left(i\right)+0.5\right)$$
(4) Time series $Z_{k}^{m,c}\left(j\right)=\left\{z_{k}^{c}\left(j\right),z_{k}^{c}\left(j+d\right),\cdots ,z_{k}^{c}\left(j+\left(m-1\right)d\right)\right\},j=1,2,\cdots ,n-\left(m-1\right)d$, (m−1) is the embedding dimension and *d* is the time delay.
(5) Each time series $Z_{k}^{m,c}\left(j\right)$ maps to a fluctuation-based dispersion pattern $\pi_{u_{0}u_{1}\cdots u_{m-1}}$, where $z_{k}^{c}\left(j\right)=u_{0},z_{k}^{c}\left(j+d\right)=u_{1},z_{k}^{c}\left(j+\left(m-1\right)d\right)=u_{m-1}$. The number of fluctuation-based dispersion modes assignable to each time series $Z_{k}^{m,c}\left(j\right)$ is equal to ${\left(2c-1\right)}^{m-1}$.
(6) For each fluctuation-based dispersion pattern $\pi_{u_{0}u_{1}\cdots u_{m-1}}$, the relative frequency is obtained by Equation (12).
  (12) $$W\left(\pi_{v_{0}\cdots v_{m-1}}\right)=\frac{\#\{j|j\leq n-\left(m-1\right)d,Z_{k}^{m,c}\left(j\right)hastype\pi_{v_{0}\cdots v_{m-1}}\}}{n-(m-1)d}$$
  where # means cardinality.
(7) The Refined Composite Multiscale Fluctuation-based Dispersion Entropy (RCMFDE) is obtained by the following formula:
  (13) $$RCMFDE=-\sum_{\pi =1}^{{\left(2c-1\right)}^{m-1}}\sum_{k=1}^{\tau }W\left(\pi_{v_{0}\cdots v_{m-1}}\right)\times \ln(\sum_{k=1}^{\tau }W(\pi_{v_{0}\cdots v_{m-1}})).$$

The RCMFDE algorithm has four parameters, which are the embedding dimension *m*, the class number *c*, the delay time *d*, and the maximum scale factor $\tau_{\max}$. A study [23] pointed out that the results of the RCMFDE do not change significantly with the time delay *d*, and a different embedding dimension *m* and class number *c* have influence on RCMFDE. The higher the number of dispersion modes based on potential fluctuations (ln((2c−1)m−1)), the higher the RCMFDE value [23]. When *m* and *c* are too small, the ability of RCMFDE to detect signal mutations is lower, but the larger *m* and *c* are, the longer the algorithm runs. For samples of the same category, the feature vectors should be as similar as possible; for samples of different categories, the feature vectors should be significantly different. If the parameter selection is not suitable, it may cause instability of entropy value, incomplete extraction of hidden feature information or excessively long operation time, rendering it difficult to classify correctly. Therefore, it is necessary to select appropriate values of the class number *c* and the embedding dimension *m*.

### 3.2. Genetic Algorithm

Genetic Algorithm (GA) is a computational model that simulates the biological evolution process of natural selection and genetic mechanism of Darwin’s theory of biological evolution. It is a method to search for optimal solutions by simulating the natural evolution process [24]. When solving more complex combinatorial optimization problems, this algorithm can usually obtain better optimization results faster than some conventional optimization algorithms. The specific process of GA is as follows:

(1) Set the evolutionary generation counter *t* = 0, set the maximum evolutionary generation *T*, and randomly generate *M* individuals as the initial population P(0).
(2) Determine the fitness function and calculate the fitness of each individual in the population P(t).
(3) Apply the selection operator, the crossover operator, and the mutation operator to the population P(t), and then obtain the next generation population P(t+1).
(4) If t=T, or the change of the fitness function value reaches the given threshold, the optimal fitness individual is used as the optimal solution output. If t<T, and the change of the fitness function value is greater than the given threshold, define t=t+1, and repeat steps (2)–(4).

### 3.3. Parameter Adaptive Refined Composite Multiscale Fluctuation-Based Dispersion Entropy

**Figure 1:**
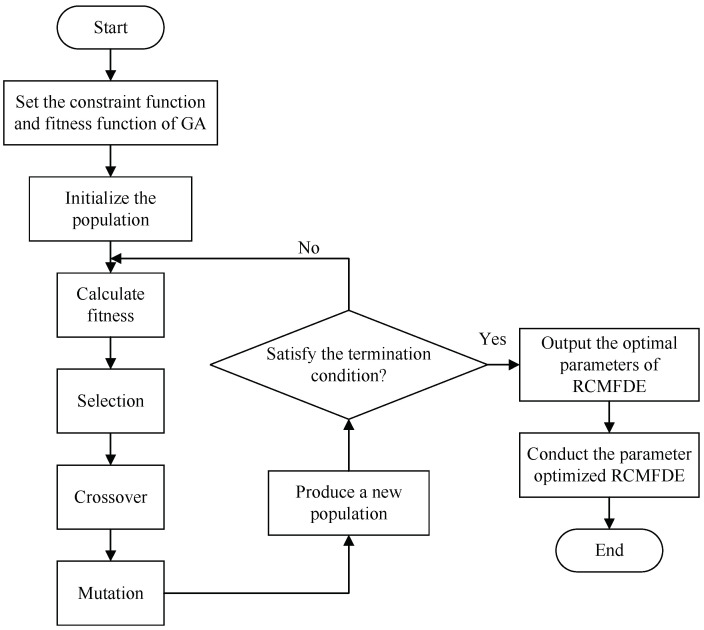
The flowchart of using GA to optimize the parameters of RCMFDE.

The settings of the embedding dimension *m* and the class number *c* of RCMFDE affect its final entropy value, entropy value stability and operation time. If the parameter settings are unreasonable, the best processing effect will not be achieved. Therefore, a suitable method is needed to adaptively select the embedding dimension *m* and the class number *c* in RCMFDE. For the above problems, this paper proposes a Parameter Adaptive Refined Composite Multiscale Fluctuation-based Dispersion Entropy (PARCMFDE). The method performs parameter optimization through Genetic Algorithm (GA) to determine the optimal parameter combination of the embedding dimension *m* and the class number *c* in RCMFDE. Figure 1 shows the flowchart of using GA to optimize the parameters of RCMFDE. The steps of parameter optimization in PARCMFDE are described as follows:

(1) Determine the approximate range and encoding length of the embedding dimension *m* and the class number *c*, and perform real encoding. The constraint function of the parameters is ${\left(2c-1\right)}^{m-1}<\left\lfloor \frac{L}{\tau_{\max}}\right\rfloor$, where *L* represents the data length, $\tau_{\max}$ is the maximum scale factor, and $\left\lfloor .\right\rfloor$ represents rounding.
(2) Initialization: Set the evolutionary generation counter t=0, set the maximum evolutionary generation *T* to 200, and randomly generate *M* individuals as the initial population P(0).
(3) Calculate the fitness of each individual in the population P(t). Skewness can characterize the overall profile of a set of data. The larger the absolute value of skewness, the more problematic the performance of the mean, and the smaller the absolute value of skewness, the more reliable the mean [25]. Therefore, this paper selects the square function of RCMFDE skewness (ske) as the fitness function and finds its minimum value. The RCMFDE at all scales of the time series $S=\{s_{1},s_{2},\cdots ,s_{n}\}$ are composed of the series $H_{P}\left(x\right)=\left\{H_{p}\left(1\right),\cdots ,H_{p}\left(m\right)\right\}$, and the skewness (ske) is calculated by the following formula:
  (14) $$ske=E{\left[H_{p}\left(X\right)-H_{p}^{m}\left(X\right)\right]}^{3}/{\left[H_{p}^{d}\left(X\right)\right]}^{3}$$
  where $H_{p}^{m}\left(X\right)$ is the mean of $H_{p}\left(X\right)$, $H_{p}^{d}\left(X\right)$ is the standard deviation of $H_{p}\left(X\right)$, and $E\left[.\right]$ represents the mathematic expectation. The fitness function is taken as $f=ske^{2}$.
(4) Apply selection operator, crossover operator and mutation operator to the population. After the population P(t) is selected, crossed and mutated, the next generation population P(t+1) is obtained.
(5) Judgment of termination condition: If t≥T, or the change of fitness function value is less than $10^{-6}$, then the individual with the smallest fitness obtained in the evolution process is used as the optimal solution, and the optimal parameter combination *m*, *c* is obtained. If t<T, and the change of the fitness function value is greater than $10^{-6}$, define t=t+1, and repeat steps (3)–(5).
(6) Use the parameter-optimized RCMFDE to extract the features of the reconstructed rotating machinery vibration signal.

### 3.4. Error Analysis and Comparison Results

**Figure 2:**
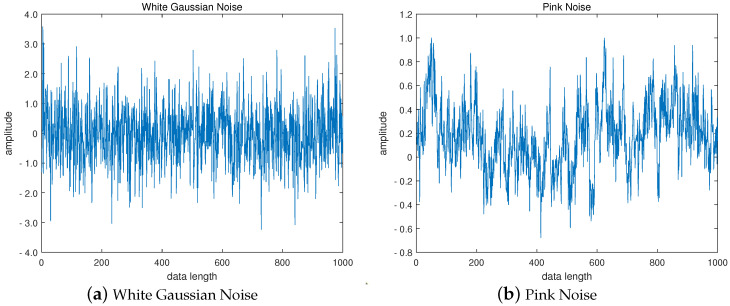
Time Domain Waveform of (**a**) White Gaussian Noise and (**b**) Pink Noise.

**Figure 3:**
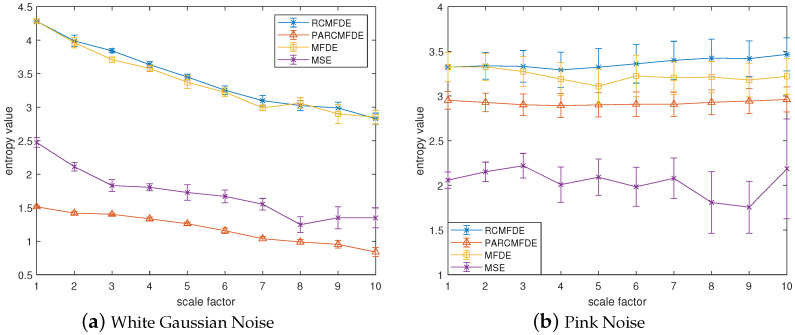
Entropy of different algorithms of (**a**) White Gaussian Noise and (**b**) Pink Noise.

To demonstrate the effectiveness of PARCMFDE in assessing the complexity and irregularity of time series, PARCMEDE of white Gaussian and pink noise signals are calculated and compared with RCMFDE, Multiscale Fluctuation-based Dispersion Entropy(MFDE), and Multiscale Sample Entropy (MSE). Furthermore, to compare the accuracy of the complexity measures with different entropies, 10 groups of white Gaussian noise and 10 groups of pink noise were randomly generated. For unified comparison, the maximum scale factor $\tau_{\max}$ of the entropy is set to 10, and the time delay *d* is set to 1. Among them, the embedding dimension *m* and the class number *c* of PARCMFDE take 2 and 3, respectively, in white Gaussian noise, and take m=2, c=21 in pink noise. Based on experience, the embedding dimension *m* and the class number *c* of RCMFDE and MFDE take the default values of m=3 and c=6, respectively. The MSE takes the default value of m=2, r=0.15×σ, and σ represents the standard deviation of the signal. Figure 2a,b show the time-domain waveforms of white Gaussian noise and pink noise. Figure 3a,b plot the error bars of different entropy algorithms for white Gaussian noise and pink noise, respectively. The entropy value of pink noise time series should remain almost constant, while the entropy value of white Gaussian noise data should decrease monotonically [26]. It can be seen from Figure 3a that with the increase of the scale factor τ, the average curves of the four entropies of white Gaussian noise (i.e., RCMFDE, PARCMFDE, MFDE and MSE) bear a downward trend which indicates that the four algorithms have good sensitivity in detection complexity. Furthermore, the standard deviation of PARCMFDE for white Gaussian noise at each scale is smaller than that of RCMFDE, MFDE and MSE, indicating that PARCMFDE has higher accuracy than the other three algorithms on the complexity measure of white Gaussian noise. It can be seen from Figure 3b that the MSE of pink noise exhibits a slight downward trend with large fluctuations, but the PARCMFDE remains almost unchanged, indicating that the RCMFDE is better than the MSE. Furthermore, the standard deviation of PARCMFDE for pink noise at each scale is smaller than that of RCMFDE, MFDE and MSE, indicating that PARCMFDE can provide a more accurate complexity estimate for pink noise [27]. That is, PARCMFDE is effective in complexity measurement and feature extraction of nonstationary signals. The standard deviation of MSE is much larger than the other three methods, indicating that MSE is insufficiently accurate regarding the complexity measurement and feature extraction of nonstationary signals.

## 4. Fuzzy C-Means Clustering

Fuzzy C-means (FCM) clustering algorithm is the most widely used fuzzy clustering algorithm based on objective function. It obtains the membership degree of each sample point to all class centers by optimizing the objective function, so as to determine the class of the sample point to achieve the purpose of automatically classifying the sample data [28].

Let $R=\{r_{1},r_{2},\cdots ,r_{n}\}$ be the set of data samples, and *n* is the number of samples. $C=\{c_{1},c_{2},\cdots ,c_{t}\}$ is the cluster center vector, and *t* is the total number of clusters. The FCM clustering algorithm minimizes the objective function shown in Equation (15) through continuous iteration of the least squares method, and its constraints are shown in Equation (16).

(15) $$J_{m}=\sum_{i=1}^{t}\sum_{k=1}^{n}{\left[\mu_{ik}\left(r_{k}\right)\right]}^{m}\left|\right|r_{k}-c_{i}\left|\right|$$

(16) $$\sum_{i=1}^{t}\mu_{ik}\left(r_{k}\right)=1$$

where $r_{k}$ is the *k*th sample point to be clustered, $\mu_{ik}$ is the degree of membership of $r_{k}$ to the *i*th cluster center $c_{i}$, *m* is the weight index of the degree of membership, generally m=2.

The cluster center $c_{i}$ and the membership matrix $\mu_{ik}$ are randomly selected initially. Then iteratively calculate through Equations (17) and (18), and stop until the change of the objective function is less than the threshold.

(17) $$C_{i}=\frac{\sum_{k=1}^{n}r_{k}\mu_{ik}^{2}}{\sum_{k=1}^{n}\mu_{ik}^{2}},i=1,2,\cdots ,t$$

(18) $$\mu_{ik}=\frac{1}{\sum_{j=1}^{t}\frac{\left|\right|r_{k}-c_{i}{\left|\right|}_{2}^{2/(m-1)}}{\left|\right|r_{k}-c_{j}{\left|\right|}_{2}^{2/(m-1)}}}$$

The average fuzzy entropy *E*, classification coefficient *S* and classification accuracy Acc are used to analyze and evaluate the clustering effect of the fuzzy C-means, which are, respectively, defined as:

(19) $$E=-\frac{1}{n}\sum_{i=1}^{t}\sum_{k=1}^{n}\mu_{ik}\ln\mu_{ik}$$

(20) $$S=\frac{1}{n}\sum_{i=1}^{t}\sum_{k=1}^{n}\mu_{ik}^{2}$$

(21) $$Acc=\frac{1}{n}\left(\sum_{i=1}^{n}1\{R_{v}=={\hat{R}}_{V}\right\})$$

where $R_{v}$ and ${\hat{R}}_{V}$ denote the actual class and the class assigned by FCM on the test dataset, *n* is the number of samples in the test dataset.

The ambiguity of clustering is represented by the average fuzzy entropy *E*, which reflects the distribution characteristics of the clustering dataset, so it can be used as an index to judge the clustering effect and correctness. The smaller the ambiguity, the higher the order of the system. The classification coefficient *S* measures the overlap between clusters, and the closer it is to 1, the more effective the clustering result [29]. Therefore, the closer *E* is to 0, the closer *S* is to 1, and the closer Acc is to 100%, the better the sample clustering effect is.

## 5. Proposed Fault Diagnosis Method

In order to quickly and reliably extract the characteristic information of the vibration signal and realize the automatic classification of the working state of the rotating machinery, a new fault diagnosis method of the rotating machinery based on FIF-PARCMFDE and Fuzzy C-means (FCM) is proposed. The specific process is as follows:

(1) Use the accelerometer to collect the original vibration signal y(x) of the rotating machinery in different states.
(2) The FIF algorithm decomposes the collected vibration signal y(x) to obtain a series of IMFs.
(3) Calculate the correlation coefficient of each order *IMF*, and select components with a correlation coefficient greater than 0.4 for reconstruction.
(4) The PARCMFDE of the reconstructed signal S(x) is calculated, and the corresponding entropy value is used as the characteristic information reflecting the working state of the rotating machinery.
(5) Input the training set into FCM to obtain the cluster centers.
(6) Input the testing set and cluster centers into FCM to automatically classify the working state of rotating machinery.

**Figure 4:**
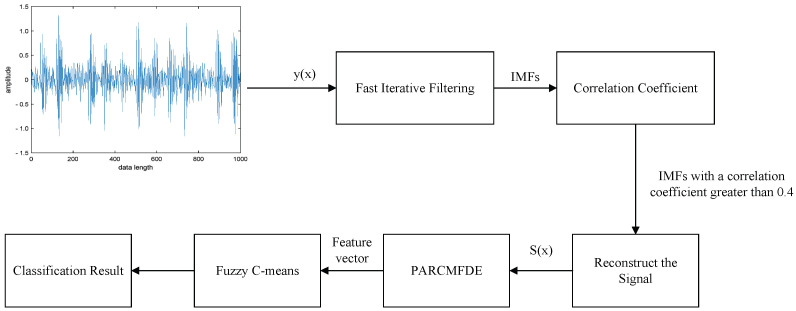
Block diagram of the proposed fault diagnosis method.

The block diagram of the proposed fault diagnosis method is shown in Figure 4.

## 6. Experimental Verification

In this section, we apply the proposed fault diagnosis method to the bearing vibration signal of Case Western Reserve University and centrifugal pump vibration signals obtained by building a water circulation experimental system. It is compared with some similar commonly used methods to evaluate the effectiveness and superiority of our method.

### 6.1. Experiment 1: Bearing Data From Cwru

#### 6.1.1. Experimental Setup

The experimental data adopts the vibration signal from the Bearing Data Center of Case Western Reserve University [30]. Vibration data are collected using accelerometers, which are mounted on the drive end bearing housing. The outer race, rolling element and inner race of rolling bearings are machined using electric sparks to simulate single point crack failure of the outer race, rolling element and inner race. The selected test bearing model is 6205-2RS, the rotational speed is 1750 r/min, and the sampling frequency is the vibration data of 12 kHz. Analyze the vibration data of normal, inner ring failure, outer ring failure, and rolling element failure. Twenty samples for each of the four bearing conditions were obtained through a non-overlapping sliding window of length 5500 points, that is, each sample contained 5500 points. The first 10 samples for each of the four bearing conditions are selected as the training set, and the remaining 10 samples for each of the four bearing conditions are selected as the testing set.

#### 6.1.2. Comparison And Analysis

To verify the effectiveness of the FIF-PARCMFDE-FCM method for bearing fault diagnosis, under the same test conditions, the vibration data of four operating states of normal bearing, inner race fault, rolling element fault and outer race fault were classified and identified.

**Figure 5:**
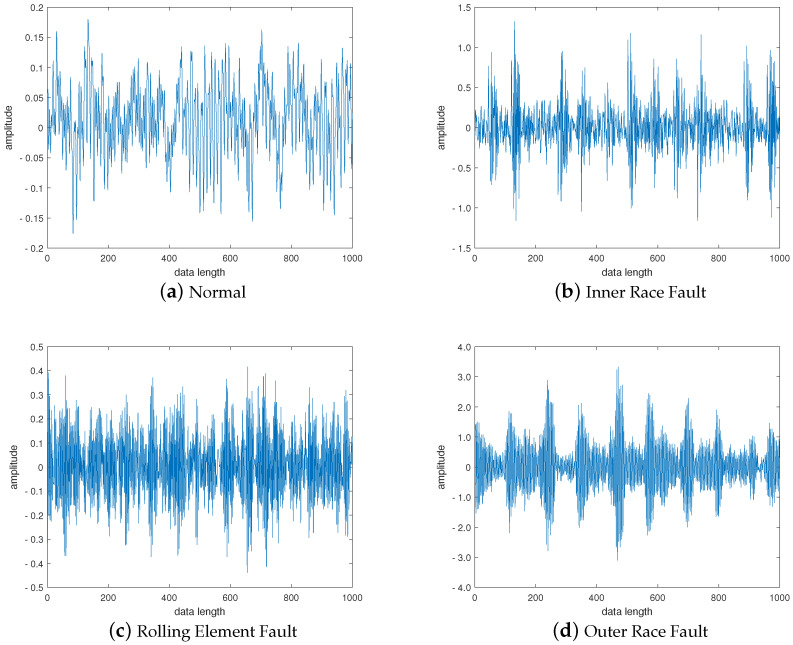
Time domain waveform of vibration signal under (**a**) Normal, (**b**) Inner Race Fault, (**c**) Rolling Element Fault and (**d**) Outer Race Fault of bearing.

The first 1000 points of the original vibration signal in the four states of the bearing are selected, as shown in Figure 5. It can be seen from Figure 5 that the vibration signals in the four states of the bearing are quite different and bear distinct characteristics, but they are not enough to be directly classified according to the waveform.

FIF decomposes the vibration signals in the four states of the bearing, and selects the first five-order *IMF* components for comparison. The *IMF* components with larger correlation coefficients can well retain the fault characteristic information of the signals [31]. The correlation coefficient between the first five-order *IMF* components and the original signal is shown in Table 1, and the component with the correlation coefficient greater than 0.4 is selected to reconstruct the signal. Therefore, the outer race fault selects IMF1 as the reconstruction signal, the inner race fault and rolling element fault select IMF1 and IMF2 for reconstruction, and the normal signal selects IMF1, IMF2 and IMF4 for reconstruction.

**Figure 6:**
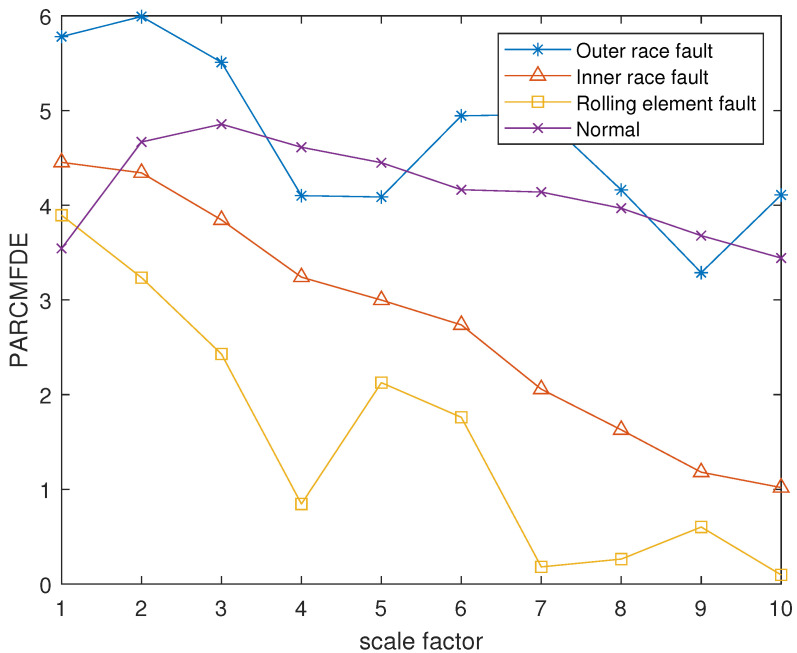
PARCMFDE in different states of bearing.

**Figure 7:**
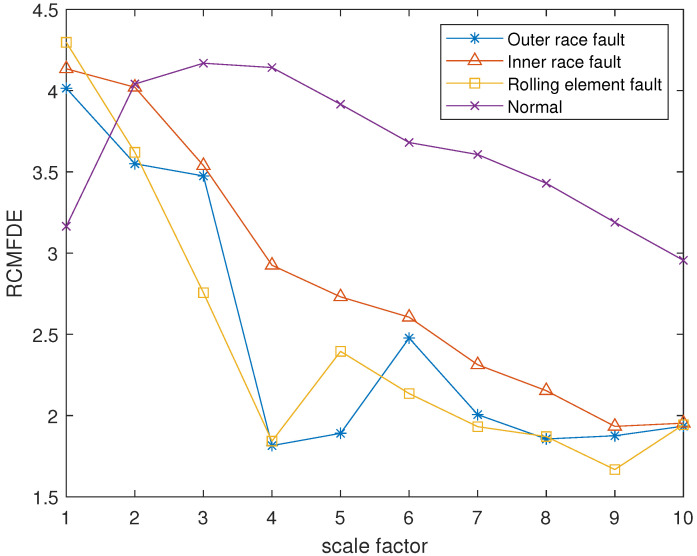
RCMFDE in different states of bearing.

**Figure 8:**
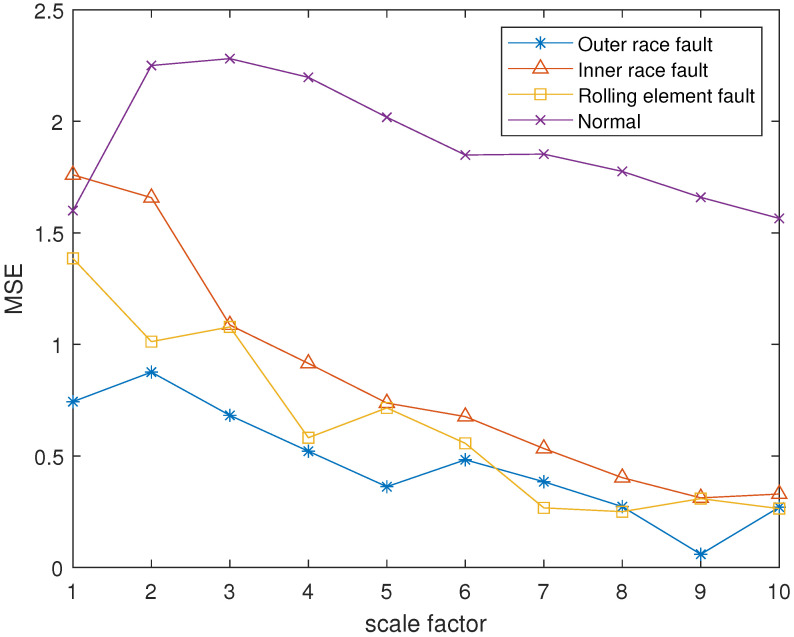
MSE in different states of bearing.

The reconstruction signals *s* of different states of the bearing are selected. The skewness is used as the objective function in GA, and set the maximum evolutionary generation *T* to 200, the threshold for the fitness function to change is $10^{-6}$. The parameters of RCMFDE are optimized by GA. Calculate the PARCMFDE, RCMFDE and MSE of the reconstructed signal *s*, where the scale factor is 10, and the embedding dimension *m* and class number *c* of PARCMFDE under different conditions are shown in Table 2. Based on experience, RCMFDE takes default values m=3, c=6, MSE takes default value m=2, r=0.15×σ, σ represents the standard deviation of the signals *s*. It can be seen from Figure 6, Figure 7 and Figure 8 that PARCMFDE, RCMFDE and MSE can all distinguish the four states of the bearing, indicating that the three methods can effectively extract the hidden features of different states of the bearing. However, compared with RCMFDE and MSE, PARCMFDE can distinguish the four states of the bearing more clearly, and is more suitable for further classification of bearing faults as a feature vector.

**Figure 9:**
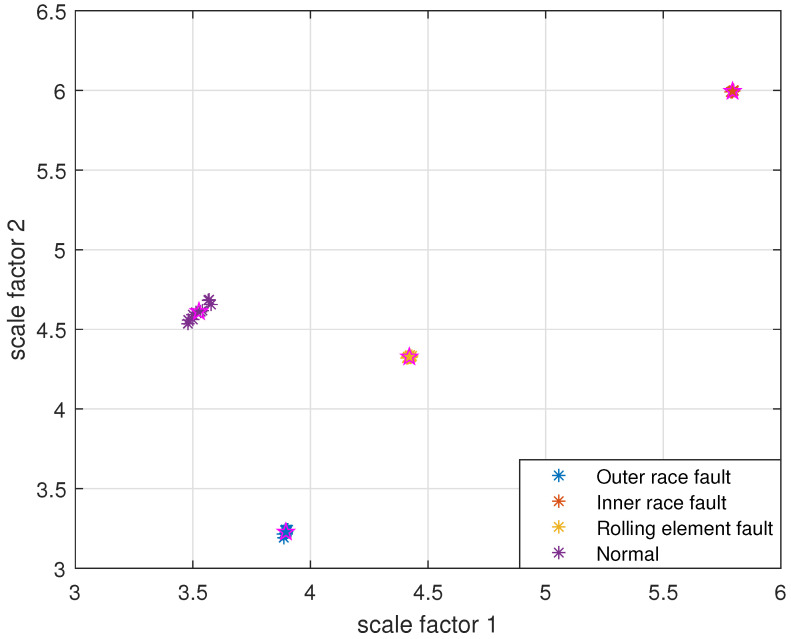
FIF-PARCMFDE-FCM clustering results of different bearing states.

**Figure 10:**
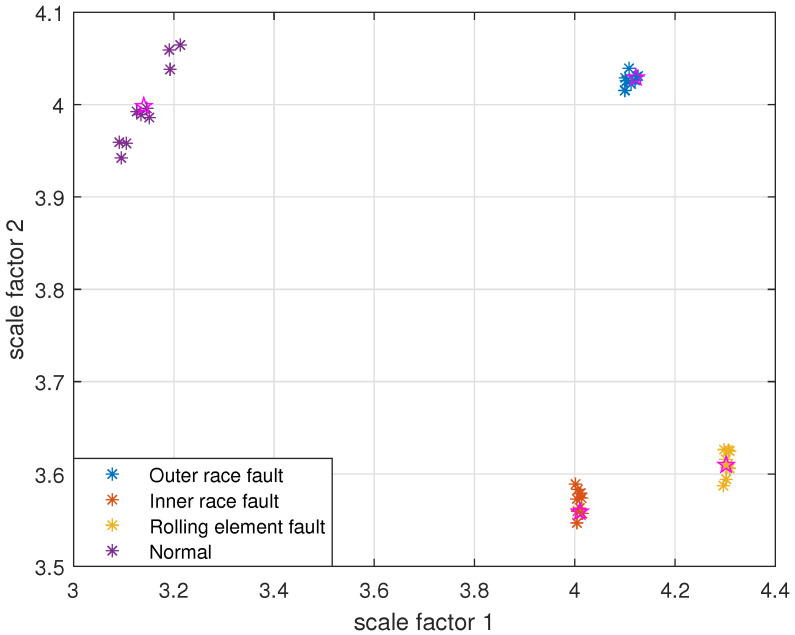
FIF-RCMFDE-FCM clustering results of different bearing state data.

**Figure 11:**
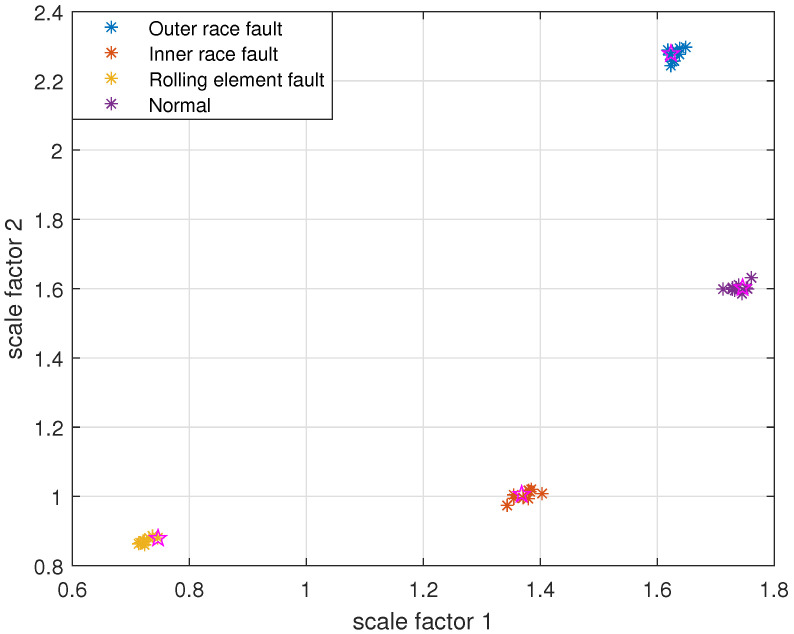
FIF-MSE-FCM clustering results of different bearing state data.

Take PARCMFDE, RCMFDE and MSE as the eigenvector matrix. Perform FCM cluster analysis on the eigenvector matrix of the training samples, and four cluster centers can be obtained. Then the obtained cluster centers and testing sample eigenvector matrix are input into FCM clustering. The clustering results are shown in Figure 9, Figure 10 and Figure 11.

It can be seen from Figure 9, Figure 10 and Figure 11 that the data points of the same state are concentrated around their respective cluster centers, and the data points of different states are separated from each other. In addition, the positions of the cluster centers obtained by different methods are different, and the degree of closeness of the data points distributed around the cluster centers is also different. In comparison, FIF-PARCMFDE-FCM have the best clustering effect, that is, the categories are most distinct, the clustering centers of various signals are far apart, and the data points of various types are compactly clustered around the clustering centers. Compared with FIF-RCMFDE-FCM and FIF-MSE-FCM, the class center distance of FIF-PARCMFDE-FCM method is larger, and the different signals are more clearly distinguished, indicating that the method has a better classification effect on various fault signals of rolling bearings.

The classification coefficient *S*, the average fuzzy entropy *E* and the classification accuracy Acc of each clustering result were calculated, respectively, and the clustering effect of the above three algorithms and the fault recognition rate were quantitatively compared. The clustering results of the above three algorithms are shown in Table 3. It can be seen from Table 3 that the classification accuracy Acc of the above four methods are all 100%. The classification coefficient *S* of the FIF-MSE-FCM method is 0.9913, the average fuzzy entropy *E* is 0.0306, and the clustering effect is poor. The classification coefficient based on FIF-PARCMFDE-FCM method is 0.9967, the average fuzzy entropy is 0.0123, and the clustering effect is the best, indicating that this method can achieve a more accurate and reliable fault diagnosis.

### 6.2. Experiment 2: Experimental Data of Centrifugal Pump

#### 6.2.1. Experimental Setup

**Figure 12:**
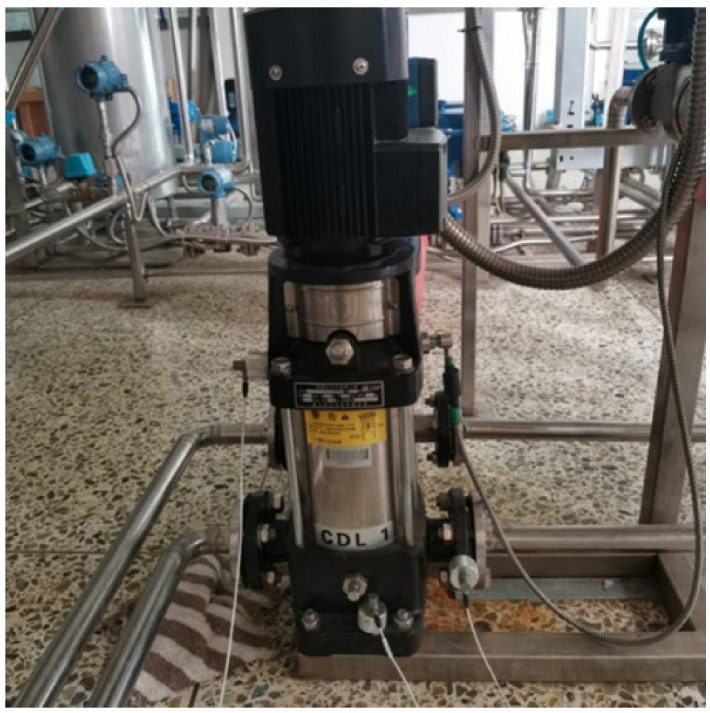
Layout of measuring points of centrifugal pump.

To verify the effectiveness of the method, a water circulation system was built in Wuhan University of Technology, and the vibration signals of centrifugal pumps in different states were collected. In this experiment, a model CDL1-11FSWPG light-duty vertical multistage centrifugal pump was selected, with a rated speed of 2900 r/min, a lift of 61 m, and a rated flow of 1 m^3^/h. According to GBT-29531-2013 pump vibration measurement and evaluation method, the vibration sensor measurement points of centrifugal pump are arranged, and vibration data in three directions of *x*, *y*, and *z* are collected at the same time. The measuring point in the *x*-axis direction is arranged on the pump casing, the measuring point in the *z*-axis direction is arranged on the base, and the measuring point in the *y*-axis direction is arranged at the outlet flange. The structure of the pump body and the arrangement of measuring points are shown in Figure 12.

**Figure 13:**
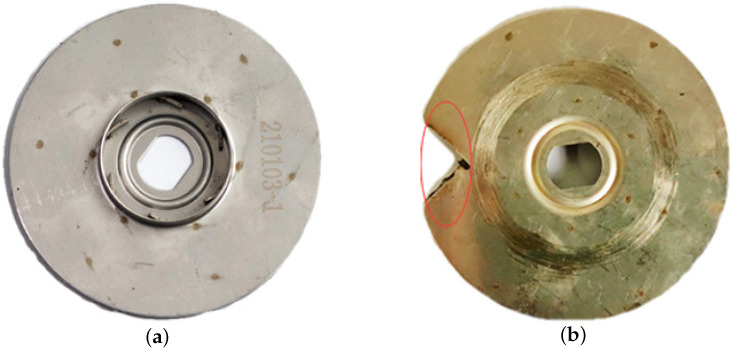
Rotor unbalance fault setup: (**a**) Impeller in normal condition, (**b**) Impeller in damaged condition.

**Figure 14:**
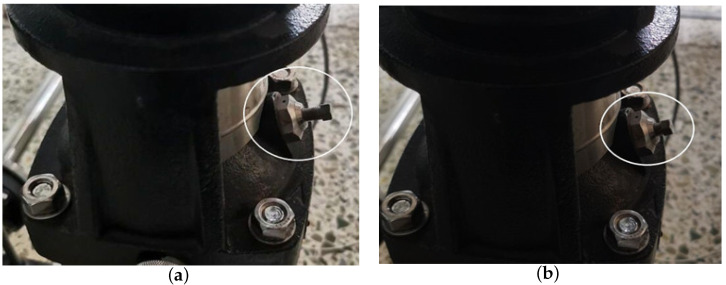
Air binding fault setting: (**a**) Exhaust screw loose, (**b**) Exhaust screw tightened.

According to the actual situation during operation of the centrifugal pump, there are rotor unbalance and air binding faults. The impeller is the main component of the rotor in the centrifugal pump. During actual operation, the impeller is in contact with the working medium, thus it is the rotor part most prone to failure. The laboratory conditions will simulate a centrifugal pump rotor unbalance fault with impeller damage, as shown in Figure 13. The centrifugal pump is not filled with the liquid to be conveyed before starting, or air will infiltrate the pump during operation, because the density of the gas is less than the density of the liquid, the centrifugal force generated is small, and the air cannot be expelled. The negative pressure generated by the fluid in the pump casing during centrifugal motion with the motor is not enough to suck the liquid into the pump casing, which is called the air binding phenomenon of the centrifugal pump. In this experiment, by tightening the exhaust screw of the centrifugal pump, and then removing the centrifugal pump, the air can enter the inner chamber of the centrifugal pump. After installing the centrifugal pump, the residual air cannot be discharged from the centrifugal pump through the exhaust screw, so as to set the air binding fault of the centrifugal pump, as shown in Figure 14.

After building the experimental platform, the sampling frequency was set to 1 kHz, the motor speed to 1015 r/min, and the acquisition system developed by Labview was employed to collect the vibration signals of the centrifugal pump in normal state. Then, the vibration signals of rotor unbalance and air bind state of the centrifugal pump were collected. A total of 25 samples for each of the three conditions of the centrifugal pump were obtained through a non-overlapping sliding window of length 4000 points. This means there are 4000 points per sample. The first 10 samples for each of the three conditions of the centrifugal pump are selected as the training set, and the remaining 15 samples for each of the three conditions are selected as the testing set.

#### 6.2.2. Comparison and Analysis

To verify the superiority of FIF-PARCMFDE-FCM clustering for fault diagnosis of centrifugal pump, under the same test conditions, the vibration data of three operating states of centrifugal pump normal, rotor unbalance fault and air binding fault were classified and identified. Furthermore, these data were compared with the cluster analysis results of FIF-RCMFDE-FCM and FIF-MSE-FCM.

**Figure 15:**
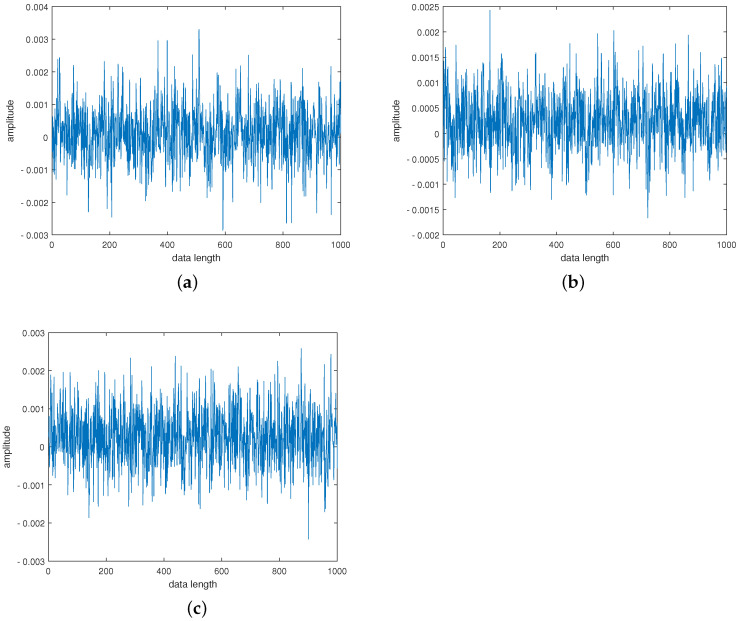
Time domain waveform of vibration signal of centrifugal pump in (**a**) Normal, (**b**) Air Bind Fault and (**c**) Rotor Unbalance Fault.

The first 1000 points of the original vibration signal in the three states of the centrifugal pump are selected, as shown in Figure 15. It can be seen from Figure 15 that the characteristics of the vibration signals in the three states of the centrifugal pump are not clear and cannot be directly classified according to the waveform. The vibration signals of the centrifugal pump in three states were decomposed by FIF, and the first five-order *IMF* components were selected for comparison. The *IMF* components with larger correlation coefficients can well preserve the fault characteristic information of the signal. The correlation coefficients between the first five-order *IMF* components and the original signal are shown in Table 4. The components with a correlation coefficient greater than 0.4 are selected to reconstruct the signal. Therefore, IMF1 and IMF2 are selected for reconstruction for normal, rotor unbalance fault and air bind fault.

**Figure 16:**
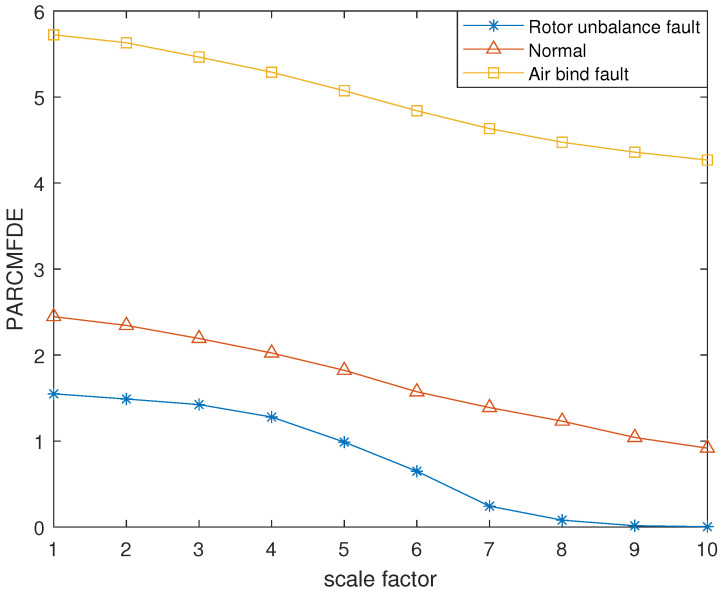
PARCMFDE in different states of centrifugal pump.

**Figure 17:**
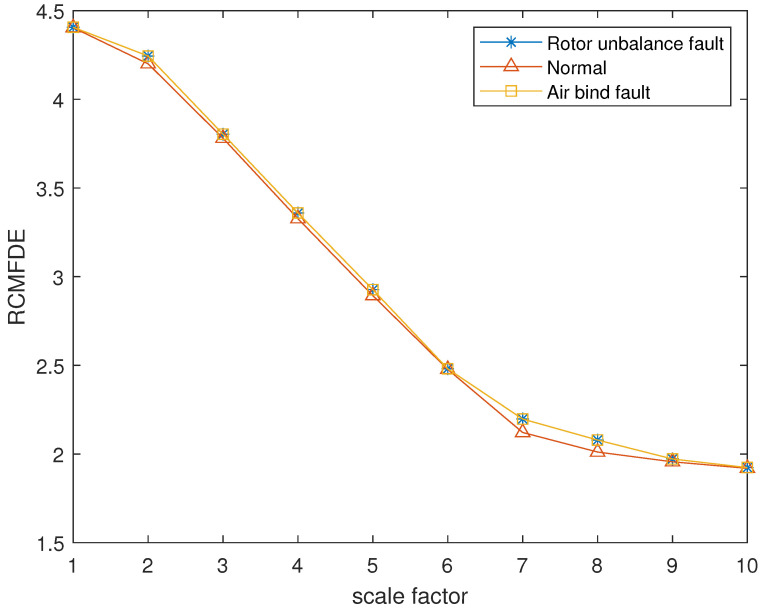
RCMFDE in different states of centrifugal pump.

**Figure 18:**
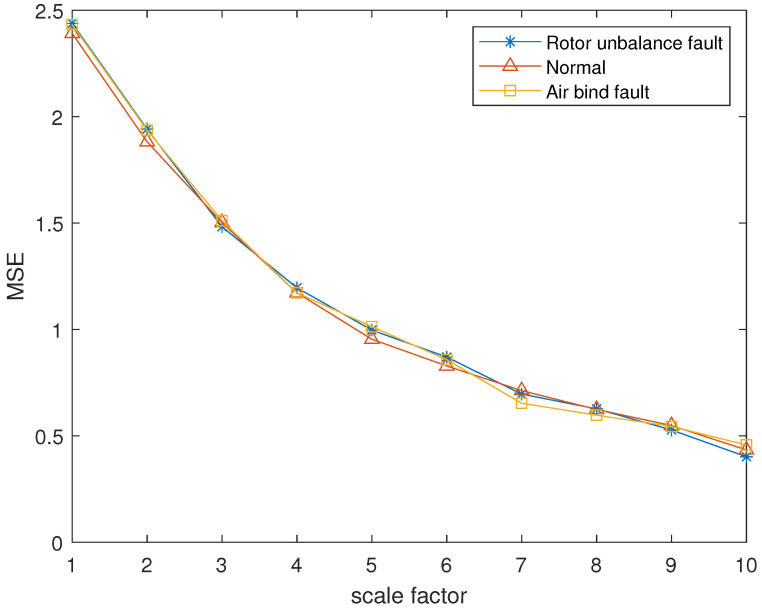
MSE in different states of centrifugal pump.

The reconstructed signals of different states of the centrifugal pump are selected. The skewness is used as the objective function in GA and the maximum evolutionary generation *T* is set to 200, the threshold for the fitness function to change is $10^{-6}$. The parameters of RCMFDE are optimized by GA. The PARCMFDE, RCMFDE and MSE of the reconstructed signal *s*, are calculated where the scale factor is 10. The embedding dimension *m* and the class number *c* of PARCMFDE in different situations are shown in Table 5. Based on experience, RCMFDE takes default values m=3, c=6, MSE takes default values m=2, r=0.15×σ, σ represents the standard deviation of signal *s*. As shown in Figure 16, PARCMFDE can clearly separate the three states of the centrifugal pump, indicating that PARCMFDE can effectively extract the hidden features of the three states of the centrifugal pump, which is suitable as a feature vector to further classify the states of the centrifugal pump. As shown in Figure 17 and Figure 18, RCMFDE and MSE are almost inseparable from the three states of the centrifugal pump, indicating that RCMFDE and MSE may not be able to effectively extract the small fault features hidden in rotating machinery, and are not suitable for further classification of centrifugal pump states as feature vectors.

**Figure 19:**
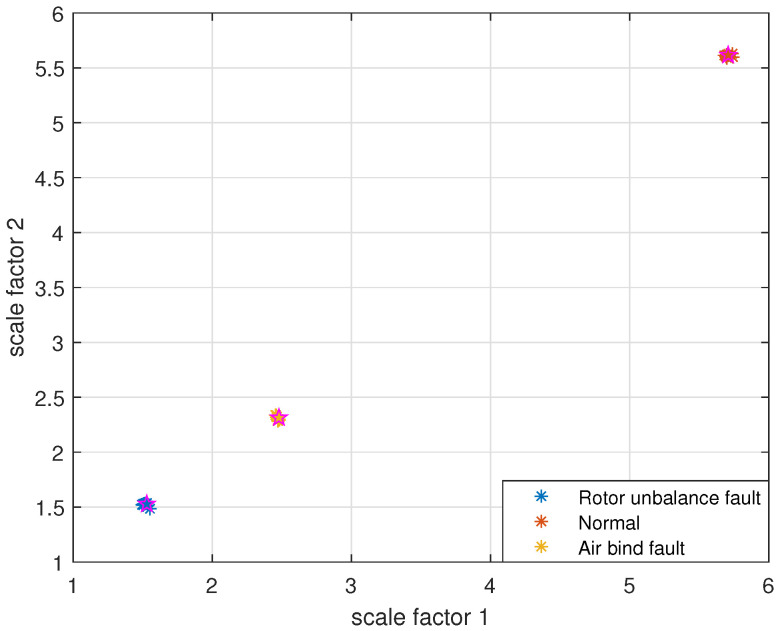
FIF-PARCMFDE-FCM clustering results of centrifugal pump data in different states.

**Figure 20:**
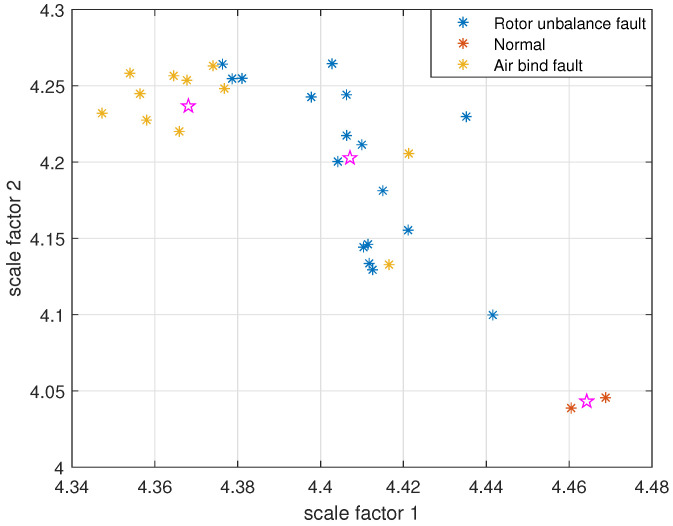
FIF-RCMFDE-FCM clustering results of centrifugal pump data in different states.

**Figure 21:**
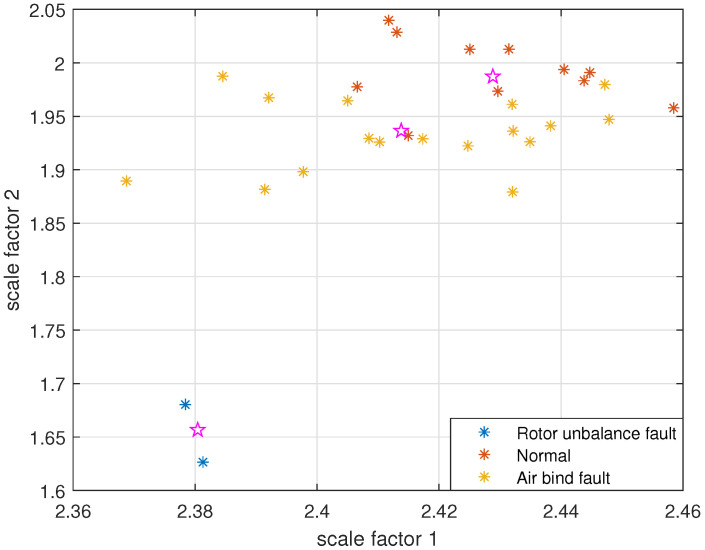
FIF-MSE-FCM clustering results of centrifugal pump data in different states.

Taking PARCMFDE as the eigenvector matrix. Perform FCM cluster analysis on the eigenvector matrix of the training samples, and three cluster centers can then be obtained. Next, the obtained cluster centers and testing sample eigenvector matrix are input into FCM clustering. The clustering results are shown in Figure 19. Similarly, RCMFDE and MSE are separately input into FCM clustering as eigenvector matrices. The clustering results are shown in Figure 20 and Figure 21.

The clustering effect and classification accuracy of the above three algorithms are quantitatively compared, and the classification coefficient *S*, average fuzzy entropy *E* and classification accuracy Acc of each method are calculated, respectively, as shown in Table 6. From Figure 19 and quantitative analysis, it can be seen that FIF-PARCMFDE-FCM clearly distinguished the fault categories, the cluster centers of various signals are far apart, and the data points of various types are compactly clustered around the cluster centers. Moreover, the classification accuracy Acc is 100%. This shows that the method has a good classification effect on the fault signals of the centrifugal pump in different states. However, the methods based on FIF-RCMFDE-FCM and based on FIF-MSE-FCM exhibit poor clustering effect, serious misclassification, and low classification accuracy Acc.

It can be seen from the above two experiments that the accuracy of the three methods in experiment 1 is 100%, but the accuracy of FIF-MSE-FCM and FIF-RCMFDE-FCM is greatly reduced in Experiment 2. There are two reasons: 1. The vibration signal fault feature of Experiment 1 are more evident and easy to distinguish. However, the fault features of the vibration signal in Experiment 2 are relatively weak and difficult to extract accurately. 2. By using GA to optimize the parameters of RCMFDE, the problem regarding the selection of *m* and *c* depends on experience is solved, and the performance of feature extraction is improved. This reflects that the FIF-PARCMFDE-FCM can adaptively select parameter combinations according to different application scenarios, which has better adaptability for signals that are more difficult to classify and with less obvious fault features.

## 7. Conclusions

To overcome the shortcomings of traditional feature extraction methods that bear difficulty in extracting tiny fault features hidden in vibration signals, and the shortcomings of RCMFDE to select parameters based on experience, a PARCMFDE is proposed. PARCMFDE takes the skewness of RCMFDE as the objective function, and uses genetic algorithm to optimize parameters. PARCMFDE can more accurately extract tiny fault features hidden in vibration signals of rotating machinery. At the same time, a new fault diagnosis method for rotating machinery based on FIF-PARCMFDE-FCM is proposed, which can classify rotating machinery faults accurately and automatically without depending on the length of data samples. FIF quickly decomposes the original vibration signal, and selects components with large correlation coefficients for reconstruction. The reconstructed signal features are extracted by PARCMFDE, and the feature vector is formed into FCM for automatic label-free classification. The bearing experiments with clear fault characteristics prove that the classification performance of this method is superior to other methods. Experiments on centrifugal pumps with weak fault features demonstrate that this method can extract hidden weak fault features from vibration signals and perform accurate and reliable automatic classification. Therefore, the proposed diagnostic method can achieve reliable diagnosis performance for rotating machinery.

However, the proposed method only identifies single faults of rotating machinery, and does not consider the identification of compound faults. Furthermore, PARCMFDE is slower than RCMFDE. Therefore, the identification of compound faults in rotating machinery and the improvement of the computing speed of PARCMFDE will be regarded as the focus of our future work.

## Figures and Tables

**Table 1:** Correlation coefficients of bearings in different states.

Bearing States	Correlation Coefficients				
	IMF 1	IMF 2	IMF 3	IMF 4	IMF 5
Normal	0.62	0.653	0.3923	0.5282	0.33
Outer Race Fault	0.9992	0.2421	0.0525	0.0203	0.0117
Inner Race Fault	0.9057	0.5787	0.2222	0.0509	0.0062
Rolling Element Fault	0.9708	0.4376	0.2246	0.1306	0.0661

**Table 2:** PARCMFDE parameters of bearings in different states.

Bearing Status	Embedding Dimension *m*	Class Number *c*
Outer Race Fault	2	232
Inner Race Fault	3	7
Rolling Element Fault	3	5
Normal	4	4

**Table 3:** FCM clustering effect of different bearing entropy algorithms.

Algorithms	Classification Coefficient *S*	Average Fuzzy Entropy *E*	Classification Accuracy *Acc*
FIF-PARCMFDE-FCM	0.9967	0.0123	100%
FIF-RCMFDE-FCM	0.9935	0.0239	100%
FIF-MSE-FCM	0.9913	0.0306	100%

**Table 4:** Correlation coefficients of centrifugal pumps in different states.

States	Correlation Coefficients				
	IMF 1	IMF 2	IMF 3	IMF 4	IMF 5
Normal	0.8241	0.5885	0.3266	0.3127	0.2019
Air Bind Fault	0.7979	0.5945	0.3538	0.3561	0.2545
Rotor Unbalance Fault	0.8230	0.5870	0.3244	0.2722	0.1289

**Table 5:** PARCMFDE parameters of centrifugal pump in different states.

Status	Embedding Dimension *m*	Class Number *c*
Normal	2	7
Air Bind Fault	2	192
Rotor Unbalance Fault	2	3

**Table 6:** FCM clustering effects of different entropy algorithms for centrifugal pumps.

Algorithms	Classification Coefficient *S*	Average Fuzzy Entropy *E*	Classification Accuracy *Acc*
FIF-PARCMFDE-FCM	0.9933	0.0215	100%
FIF-RCMFDE-FCM	0.7819	0.3849	57%
FIF-MSE-FCM	0.6313	0.5962	63%

## Data Availability

The data used in this study are all owned by the research group and will not be transmitted.
